# Mephedrone and Nicotine: Oxidative Stress and Behavioral Interactions in Animal Models

**DOI:** 10.1007/s11064-015-1566-5

**Published:** 2015-04-11

**Authors:** Barbara Budzynska, Anna Boguszewska-Czubara, Marta Kruk-Slomka, Jacek Kurzepa, Grazyna Biala

**Affiliations:** Department of Pharmacology and Pharmacodynamics, Medical University of Lublin, 4A Chodzki Street, 20-093 Lublin, Poland; Department of Medical Chemistry, Medical University of Lublin, 4A Chodzki Street, 20-093 Lublin, Poland

**Keywords:** Mephedrone, Nicotine, Oxidative stress, Memory, Anxiety, Locomotor sensitization

## Abstract

The purpose of our experiment was to examine the influence of co-administration of nicotine and mephedrone on anxiety-like behaviors, cognitive processes and the nicotine-induced behavioral sensitization as well as processes connected with induction of oxidative stress in the brain of male Swiss mice. The results revealed that co-administration of subthreshold doses of mephedrone and nicotine (0.05 mg/kg each) exerted marked anxiogenic profile in the elevated plus maze and displayed pro-cognitive action in the passive avoidance paradigm (nicotine 0.05 mg/kg and mephedrone 2.5 mg/kg). Furthermore, one of the main findings of the present study was that mephedrone, administered alone at the dose not affecting locomotor activity of mice (1 mg/kg), enhanced the expression of nicotine-induced locomotor sensitization. Moreover, mephedrone administered with nicotine decreased general antioxidant status and catalase activity as well as antioxidant enzymes activity in the hippocampus and prefrontal cortex and increased concentration of malondialdehyde, an indicator of lipid peroxidation processes. Considering the likelihood that mephedrone is taken as a part of polydrug combination with nicotine, the effects of this combination on mammalian organisms have been confirmed in our study. Understanding the consequences of co-administration of psychoactive substances on the central nervous system and oxidative processes in the brain provide the important toxicological significance, and may be useful in polydrug intoxication treatment.

**Electronic supplementary material:**

The online version of this article (doi:10.1007/s11064-015-1566-5) contains supplementary material, which is available to authorized users.

## Introduction

The use of different types of club drugs, especially by young people, has been considered an ignored phenomenon for a long time. Legal highs or club drugs, various kinds of products containing psychoactive substances, were often taken as a part of the nightclub scene with alcohol, marijuana or amphetamines. One such club drug is mephedrone (RS)-1-(4-methylphenyl)-2-metyloaminopropan-1-one (other names: 4-metylometcatynon, 4-MMC, M-CAT), a synthetic derivative of cathinone [1].

Experienced users of mephedrone have equated its powerful psychostimulant, entactogenic and hallucinogenic properties to those of cocaine, amphetamine, or MDMA (3,4-methylenedioxy-methamphetamine) [2, 3]. However, only a few experimental studies have investigated its behavioral effects on laboratory animals. The first preclinical studies of the effects of mephedrone, published by Kehr et al. [4], reported significant, rapid and dose-dependent increases in both serotonin (5-HT) and dopamine (DA) levels in rats exposed acutely to this compound. This work was extended by the finding that mephedrone causes a rapid decrease in striatal DA and hippocampal 5-HT transporters function. Furthermore, mephedrone inhibits both synaptosomal DA and 5-HT uptake [5]. Recent preclinical findings point to its affinity for the serotoninergic and the dopaminergic receptors [6] and capacity to activate mesolimbic regions [7]. Moreover, several studies have established a contradictory data concerning the neurotoxic effect of mephedrone using a binge-like regimen. Accordingly, Angoa-Pérez et al. [8–10] show that mephedrone does not cause damage of long lasting hippocampal 5-HT and DA nerve endings in mice and does not enhance the effect of MDMA on 5-HT transporters (SERT) or tryptophan hydroxylase 2 (TPH2). Therefore, Martínez-Clemente et al. [6] observed loss of dopaminergic and serotoninergic neurons. Additionally, it is well established that psychostimulant drugs, including amphetamines or cocaine, have been found to exert potent neurotoxic effects due to their ability to increase the formation of reactive oxygen and nitrogen species (ROS and RNS) as well as intensification of lipids peroxidation processes [11]. However, little is still known about oxidative mechanisms of mephedrone neurotoxicity.

Although mephedrone demonstrates a unique pharmacological profile, similarities in the mechanisms of action among mephedrone and nicotine, a natural alkaloid present in tobacco, can be observed. Nicotine exerts its effects through activation of central nicotinic acetylcholine receptors (nAChR) [12]. These receptors are widely distributed in the central nervous system (CNS), and promote the release of several neurotransmitters, such as acetylcholine (ACh), DA, noradrenalin, 5-HT and gamma-aminobutiric acid (GABA) [12]. It is well documented that nicotine exerts cognitive effects [13–15], analgesia [16], and an influence on anxiety- [17], or depression-like behaviors [18]. The effects of nicotine have been extensively investigated not only in humans, but also in animals and several cell systems [19–24].

As mentioned above, mephedrone is often used in combination with other substances, including nicotine, ethanol, marijuana, amphetamines, cocaine and caffeine. However, we observe the lack of experimental data concerning the behavioral and biochemical effects relating to the combined use of mephedrone with other psychoactive substances, although the problem seems to be significant. There are currently no studies describing the behavioral effects and influence on oxidative stress processes of acute co-administration of mephedrone and nicotine. Thus, the present study aimed to examine the interactions between mephedrone and nicotine on memory consolidation processes observed in the passive avoidance (PA) test. We also used the elevated plus maze (EPM), a standard behavioral model that can assess anxiety responses, after concomitant administration of both drugs. Additionally, the present studies were undertaken to investigate behavioral locomotor effects of these drugs. We used the nicotine-induced locomotor sensitization procedure evaluated in our previous studies [25] to examine if nicotine-experienced mice developed hyper-reactivity to the locomotor stimulating effect of mephedrone. The total antioxidant status (TAS), the activity of catalase (CAT), an antioxidant enzyme, as well as concentration of malondialdehyde (MDA) within the brain tissue were also determined to evaluate the general effects of mephedrone administered alone or with nicotine on the antioxidant barrier. Considering the likelihood that mephedrone is taken as a part of polydrug combination with nicotine, knowledge about drug interactions may be important in the treatment of this kind of addiction.

## Materials and Methods

### Animals

The experiments were carried out on naive male Swiss mice (Farm of Laboratory Animals, Warsaw, Poland) weighing 20–25 g at the beginning of experiments. The animals were maintained under standard laboratory conditions (12 h light/dark cycle, room temperature 21 ± 1 °C) with free access to tap water and laboratory chow (Agropol, Poland) and were adapted to the laboratory conditions for at least 1 week. Each experimental group consisted of 8–10 animals. All experiments were conducted according to the National Institute of Health Guidelines for the Care and Use of Laboratory Animals and to the European Community Council Directive for the Care and Use of Laboratory Animals of 24 November 1986 (86/609/EEC), and were approved by the local ethics committee. Different mice were used for each drug and time treatment.

### Drugs

The following compounds were tested: (−) nicotine hydrogen tartrate (0.05 and 0.5 mg/kg, Sigma-Aldrich, St. Louis, MO, USA) and mephedrone [(RS)-2-methylamino-1-(4-methylphenyl] propan-1-one, 0.05, 0.1, 0.25, 1, 2.5, 5 and 10 mg/kg; Toronto Research Chemicals Inc.,). Drugs were dissolved in saline solution (0.9 % NaCl), nicotine administered subcutaneously (s.c.) whereas mephedrone was administered intraperitoneally (i.p.) at a volume of 10 ml/kg. Drug doses refer to the salt form. The pH of the nicotine solution was adjusted to 7.0. Fresh drug solutions were prepared on each day of experimentation. Control groups received saline injections of the same volume and via the same route of administration.

The doses of mephedrone and nicotine were chosen based on literature data [26], our recently published articles [27, 28] and preliminary studies.

### Experimental Procedure and Treatment

#### The EPM Procedure

The experimental apparatus was shaped like a “plus” sign and consisted of a central platform (5 × 5 cm), two open arms (30 × 5 cm) and two equal-sized enclosed (30 × 5 × 15 cm) arms opposite to each other. The maze was made of dark Plexiglas, elevated to a height of 50 cm above the floor and illuminated by a dim light.

The used procedure was chosen based on our recently published data [27, 28] and similar to the method of Lister [29]. Anxiolytic activity was indicated by an increase in time spent in the open arms or in the number of entries to the open arms; anxiogenic effects were characterized by a decrease in those measures. The percentage of time spent on the open arms was calculated, just as was the percentage of entries into the open arm. Additionally, the number of enclosed arm entries was recorded as the indicator of motor activity of tested animals. The mice were divided into following groups: nicotine (0.05 mg/kg, s.c.), mephedrone (0.05, 0.1, 0.25, 1, 2.5, 5 and 10 mg/kg, i.p.), saline or mephedrone (0.05 mg/kg, i.p.) co-administered with nicotine (0.05 mg/kg, s.c.) The test was conducted 30 min after nicotine injection or 15 min after mephedrone administration.

#### The PA Procedure

The apparatus and PA procedure was described in detail in our previous article [28]. It consisted of two-compartment acrylic box with a lighted and darkened compartment. The light chamber was illuminated by a fluorescent light (8 W) and was connected to the dark chamber which was equipped with an electric grid floor. Entrance of the animal to the dark box was punished by an electric foot shock (0.2 mA for 2 s).

On the first day of training (pre-test), mice were placed individually into the light compartment and allowed to explore the light box. After 30 s, the guillotine door was raised to allow the mice to enter the dark compartment. When the mice entered the dark compartment, the guillotine door was closed and an electric foot-shock (0.2 mA) of 2 s duration was delivered immediately to the animal. The latency time for entering the dark compartment was recorded (TL1). If the mouse failed to enter the dark box within 300 s, it was placed into this dark box, the door was closed, and the electric foot shock was delivered to the animal. In this case, TL1 value was recorded as 300 s. In the subsequent trial (retention) 24 h later, the same mouse was again placed individually in the light compartment of the PA apparatus and the time taken to reenter the dark compartment was recorded (TL2). No foot-shock was applied in this trial. If the animal did not enter the dark compartment within 300 s, the test was stopped and TL2 was recorded as 300 s.

The experimental procedure involved examination of memory consolidation (the animals received injections of the substance after pre-test) [30, 31].

During the acute treatment, animals were allocated into the following drug groups: nicotine (0.05 mg/kg, s.c.), mephedrone (1, 2.5, 5 mg/kg, i.p.), saline or mephedrone (2.5 mg/kg, i.p.) co-administered with nicotine (0.05 mg/kg, s.c.). The drugs were administered immediately after pre-test (memory consolidation), and the mice were re-tested 24 h later.

#### Influence of Mephedrone on the Expression of Nicotine-Induced Locomotor Sensitization

This method was similar to that used in our previous experiments accordingly to the data indicating that nicotine produces robust locomotor sensitization in mice under our laboratory conditions [25]. During the pairing phase (days 1–9), mice received injections of saline (s.c.) or nicotine (0.5 mg/kg, s.c.) every other day for five sessions. The mice remained drug free for 1 week and, on day 16, the same groups of mice were further challenged with nicotine (0.5 mg/kg), mephedrone (1 mg/kg) or saline, respectively. Locomotor activity was recorded for 60 min during the pairing phase (days 1–9) and on the 16th day, immediately after injections. We have chosen the dose of mephedrone not influencing the locomotor activity administered alone.

### Collection of Tissues

Following the behavioral test conducted after repeated administration of mephedrone, the mice were anesthetized, decapitated and the whole brain was carefully taken out and rinsed in isotonic saline to remove blood. The prefrontal cortex and the hippocampus were rapidly dissected and were used for the study.

#### Preparation of Tissue Homogenates

The collected tissues were homogenized in 10 volumes of 20 mM Tris-HCl buffer (pH 7.4 on ice for 20 s) and centrifuged at 12,000×*g* for 10 min at 4 °C. The supernatant was collected and used for further study. TAS, activity of CAT, and MDA level were determined from these supernatants spectrophotometrically with use of HITACHI 2800 apparatus and microplate reader EPOCH.

#### Determination of Malondialdehyde Concentration (MDA)

MDA was measured by the thiobarbituric acid (TBA) reaction [32]. Briefly, 0.5 ml of tissue homogenate supernatant was mixed with 2.5 ml 1.22 M TCA in 0.6 M HCl and allowed to stand for 15 min. Then 1.5 ml of 0.9 % TBA was added and the mixture was incubated for 30 min in a boiling water bath. After cooling, 4 ml of n-butanol was added and the mixture was shaken variously. The samples were centrifuged at 1500 g for 10 min and then the absorbance of organic phase was measured at 532 nm with respect to blank (*n*-butanol alone). The concentration of MDA was read from the standard curve obtained by using malonaldehyde bis-dimethylacetal and expressed as μM of MDA/g of wet tissue.

#### Determination of Catalase Activity (CAT)

The activity of CAT in tissues homogenates was measured with use of OxiSelect Catalase Activity Assay kit (Cell Biolabs, San Diego, CA, USA). The assay was performed following the manufacturer’s instructions. The absorbance was read at 520 nm. Activities were calculated using a calibration curve and were expressed as µM/min/mg protein.

#### Determination of Total Antioxidant Status (TAS)

TAS of brain homogenates was determined with ready-to-use diagnostic kit TAS by RANDOX (Randox Laboratories Ltd., UK). The method assumes that ABTS^®^ [2,2′-Azino-di-(3-ethylbenzthiazoline sulphonate)] produces a radical cation ABTS^®*+^ when incubated with a peroxidase (metmyoglobin) and H_2_O_2_. The radical cation has a relatively stable blue-green color, however its production can be suppressed by the addition of antioxidants present in the examined samples. Changes in absorption measured at 600 nm are proportional to the antioxidant concentration in the tissues homogenates. Results are expressed in mM Fe/ml tissue.

### Statistical Analysis

The data were expressed as the mean ± standard error of the mean (SEM). The statistical analyses were performed by the two-way or one-way analysis of variance (ANOVA). Post hoc comparison of means was carried out with the Tukey’s test for multiple comparisons, when appropriate. The confidence limit of *p* < 0.05 was considered statistically significant.

For the memory related behaviors, changes in the PA performance were expressed as the difference between retention and training latencies and were taken as an index of latency (IL). IL was calculated for each animal and reports as the ratio:

$${\text{IL}} = {\text{TL}}2 - {\text{TL}}1/{\text{TL}}1$$

TL1—the time taken to enter the dark compartment during the training, TL2—the time taken to reenter the dark compartment during the retention [33].

## Results

### Effects of Co-administration of Mephedrone and Nicotine on Anxiety-Related Processes in the EPM Test in Mice

**Fig. 1:**
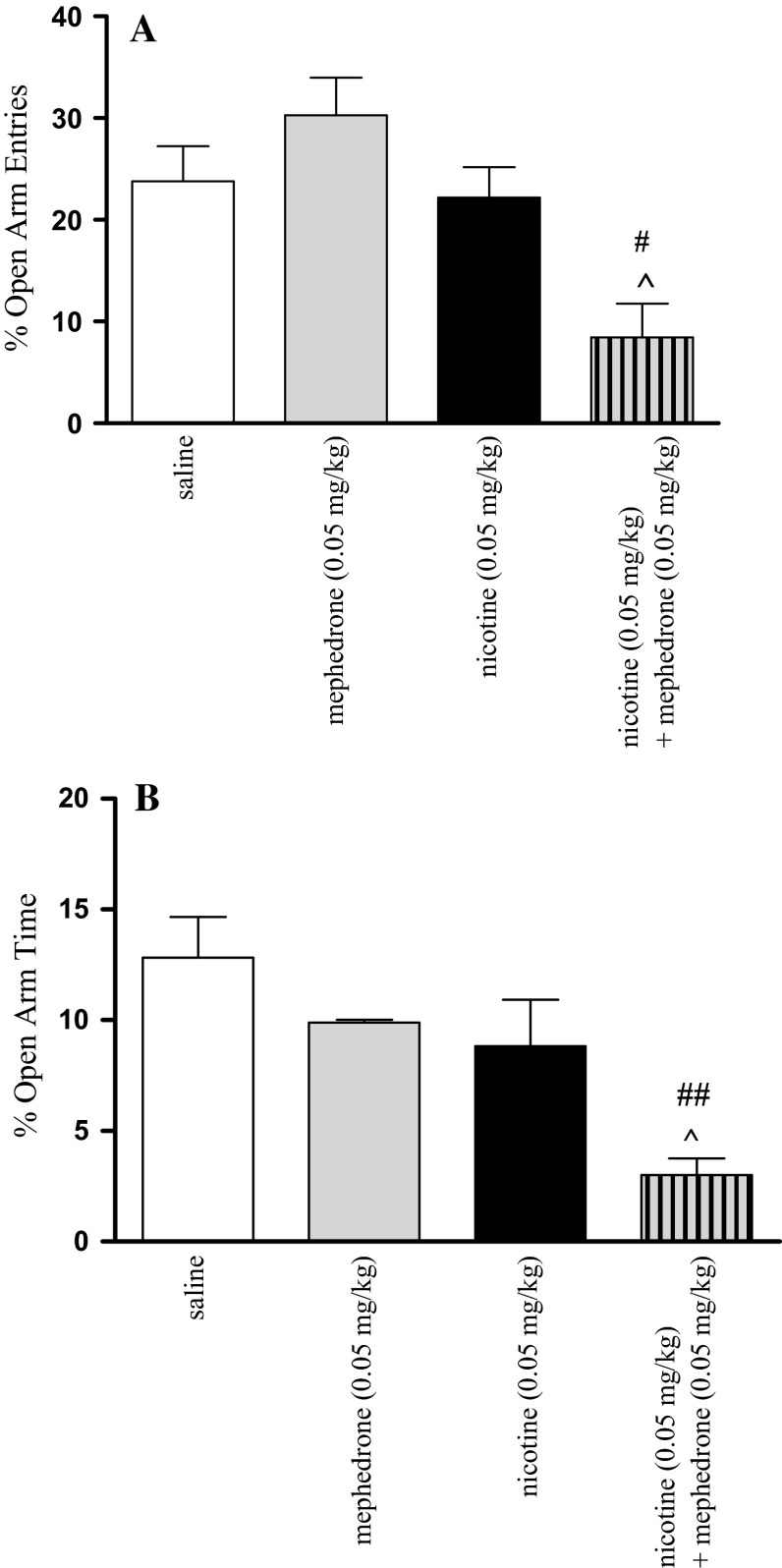
Mean (±SEM) percentage open arm entries (**a**) and percentage time spent on the open arms (**b**) in the EPM test in mice, 30 min after an acute injection of saline, mephedrone (0.05 mg/kg, i.p.), nicotine (0.05 mg/kg, s.c.), or mephedrone co-administered with nicotine; n = 8–10; ^#^ *p* < 0.05; ^##^ *p* < 0.01 versus mephedrone-treated control group; ^*p* < 0.05 versus nicotine-treated control group; Tukey’s test

Figure 1 shows that in animals which had received mephedrone (0.05 mg/kg) in combination with nicotine (0.05 mg/kg) significant effect was revealed on the percentage of open arm entries [two-way ANOVA: treatment (F(1, 34) = 12.91, *p* < 0.0010) and pre-treatment (F(1, 34) = 8.39, *p* < 0.0066), without interactions effect (F(1, 34) = 0.91, *p* = 0.3469)] (Fig. 1a) as well as of time spent on the open arms [two-way ANOVA: treatment (F(1, 33) = 11.69, *p* < 0.0017) without interactions (F(1, 33) = 8.69, *p* < 0.0591)] or pre-treatment effect [(F(1, 33) = 1.11, *p* = 0.2998)] (Fig. 1b). A post hoc analysis showed that an acute dose of nicotine (0.05 mg/kg) and mephedrone (0.05 mg/kg) alone did not affect the percentage of time spent on the open arms and of open-arm entries. However, the post hoc Tukey’s test showed that co-administration of mephedrone and nicotine at the inactive doses decreased both values. Statistically significant decrease in open-arm entries was observed as compared with nicotine-treated (*p* < 0.05) and mephedrone-treated (*p* < 0.05) control groups (Fig. 1a). Also, statistically significant decrease in percentage of time spent on the open arms was observed as compared with nicotine-treated (*p* < 0.05) and mephedrone-treated (*p* < 0.01) control groups (Fig. 1b).

### Effects of Co-administration of Mephedrone and Nicotine on Memory Related Behaviors in the PA Test

**Fig. 2:**
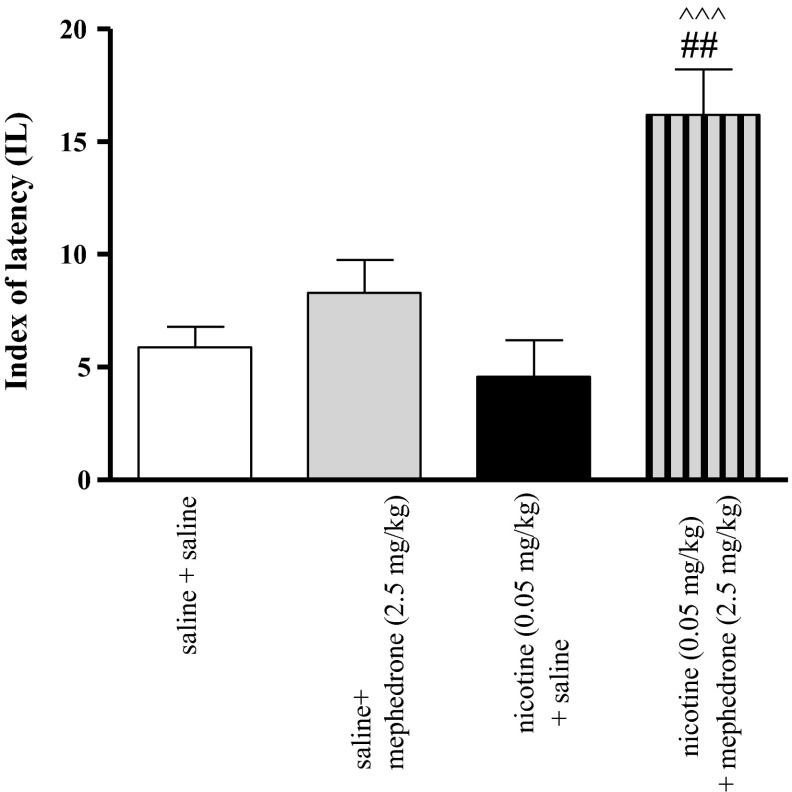
Effects of a co-administration of mephedrone (2.5 mg/kg, i.p.), nicotine (0.05 mg/kg, s.c.) or saline on memory consolidation trial using the PA test in mice. Appropriate groups of mice received compounds immediately after the pre-test. Data represent the mean ± SEM and are expressed as latency index (IL); n = 8–10; ^^^^^ *p* < 0.001 versus nicotine-treated control group; ^##^ *p* < 0.01 versus mephedrone-treated control group; Tukey’s test

Figure 2 indicates the effects of co-administration of nicotine (0.05 mg/kg, s.c.) and mephedrone (2.5 mg/kg, i.p.) on memory consolidation during the retention trial in the PA task [two-way ANOVA: pre-treatment (F(1, 28) = 4.55, *p* = 0.0419), treatment (F(2, 28) = 20.47, *p* = 0.0001) and interactions (F(2, 28) = 8.82, *p* = 0.006)]. Statistically significant improvement in memory and learning processes was observed in animals administered with subthreshold doses of nicotine (0.05 mg/kg) and mephedrone (2.5 mg/kg, see also Supplementary Fig. 7) in combination with nicotine- or mephedrone-treated mice (*p* < 0.001 and *p* < 0.01, respectively).

### Influence of Mephedrone on the Expression of Nicotine-Induced Locomotor Sensitization

**Fig. 3:**
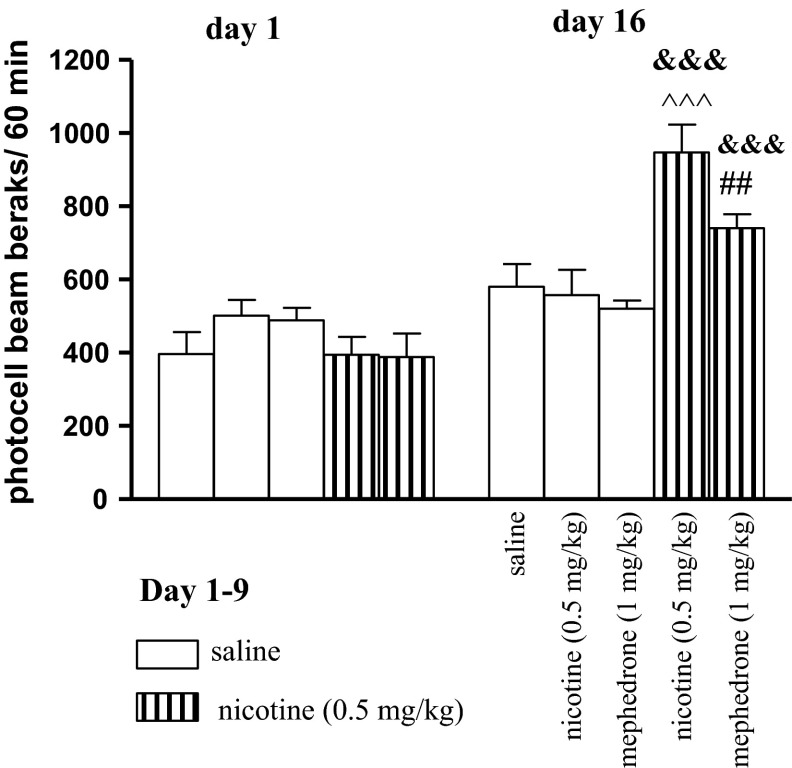
Effects of mephedrone (1 mg/kg, i.p.) on the expression of locomotor sensitization to nicotine in mice. Nicotine (0.5 mg/kg, s.c.) or saline were injected daily for 9 days, every other day; on day 16 (a test for expression of sensitization) mice were given nicotine (0.5 mg/kg), saline, or mephedrone (1 mg/kg). Data represent mean ± SEM; n = 8–10 mice per group. ^&&&^ *p* < 0.001 versus the first pairing day; ^^^^^ *p* < 0.001 versus saline-pretreated and nicotine-challenged mice; ^##^ *p* < 0.01 versus saline-pretreated and mephedrone-challenged mice; Tukey’s test

Two-way ANOVA of the locomotor response after administration of nicotine (0.5 mg/kg, s.c.) or saline during the pairing phase (day 1 and day 16—challenge) revealed a treatment effect [F(4, 69) = 3.35, *p* < 0.0111], a day effect [F(1, 69) = 46.22, *p* < 0.0001] and an interaction effect [F(4, 69) = 7.95, *p* < 0.0001] (Fig. 3). On the 1st day, one-way ANOVA did not reveal any significant treatment effect [F(1, 41) = 1.141, *p* = 0.3524]. On the 16th day, after an additional injection of nicotine, one-way ANOVA revealed a significant treatment effect [F(4, 32) = 9.236, *p* < 0.0001]. Indeed, after this last nicotine injection, a significant difference between the response was observed as compared to the 1st injection of nicotine (*p* < 0.001) or with the response to nicotine in animals repeatedly treated with saline (*p* < 0.001, Tukey’s test) (Fig. 3). Moreover, mephedrone, at the dose of 1 mg/kg injected on the day 16 to the nicotine-pretreated group significantly increased the locomotor activity of mice as compared to the first injection of nicotine (*p* < 0.001) and to the group of animals repeatedly treated with saline and challenged with mephedrone (*p* < 0.01, Tukey’s test). We have chosen the dose of mephedrone not influencing the locomotor activity administered alone as measured in the actimeter cages and the EPM paradigm as the number of enclosed arm entries (Supplementary Fig. 8; Supplementary Table 1).

### Effects of Mephedrone on Oxidative Stress Biomarkers

#### Effects of Co-administration of Mephedrone and Nicotine on Oxidative Stress Indicators

**Fig. 4:**
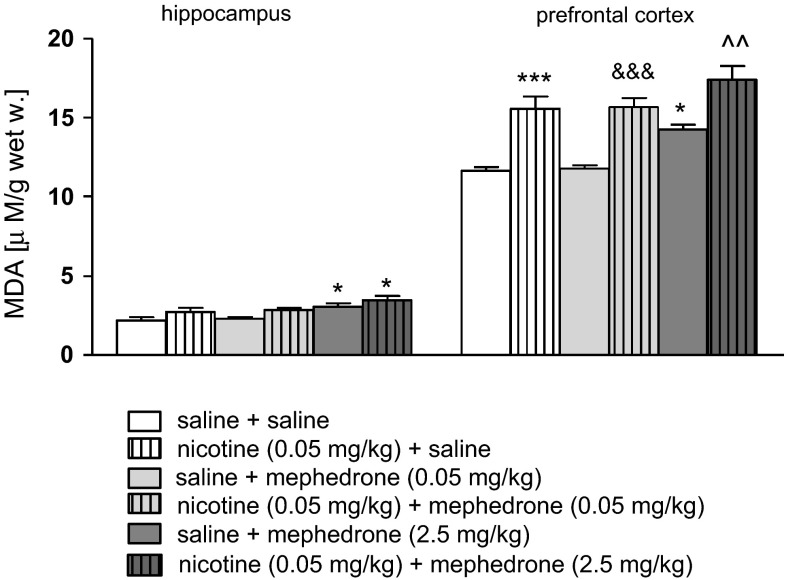
Effect of co-administration of mephedrone (0.05 or 2.5 mg/kg, i.p.) and nicotine (0.05 mg/kg, s.c.) on concentration of MDA in the hippocampus and prefrontal cortex of mice. Data are presented as the mean ± SEM; n = 8–10; *p < 0.05; ***p < 0.001 versus saline-treated control group; ^^^^ *p* < 0.01 versus mephedrone 2.5 mg/kg-treated control group; ^&&&^ *p* < 0.001 versus mephedrone 0.05 mg/kg-treated control group; Tukey’s test

The changes in value of TAS, activity of CAT and concentration of MDA were found after co-administration of mephedrone and nicotine in the examined brain structures. Figure 4 shows the activities of MDA after co-administration of mephedrone and nicotine in the hippocampus [two-way ANOVA: pre-treatment (F(1, 51) = 66.67 *p* < 0.0001), and treatment (F(2, 51) = 10.30, *p* = 0.0001), without interaction effect (F(2, 51) = 0.32, *p* = 0.7250)] and the prefrontal cortex [two-way ANOVA: pre-treatment (F(1, 50) = 9.34, *p* = 0.0036) and treatment (F(2, 50) = 9.17, *p* = 0.0004) without interaction effect (F(2, 50) = 0.10, *p* = 0.9012)]. The post hoc Tukey’s test revealed that an acute injection of mephedrone at the dose of 2.5 mg/kg significantly increased level of MDA in the hippocampus and prefrontal cortex (*p* < 0.05) versus saline-treated control group. The same effects have been seen after the dose of 5 mg/kg in both brain areas (Supplementary Fig. 9). Also, nicotine (0.05 mg/kg) significantly increased the concentration of the marker of lipids peroxidation processes in the prefrontal cortex (*p* < 0.001). Moreover, co-administration of nicotine (0.05 mg/kg) and mephedrone in both used doses (0.05 and 2.5 mg/kg) led to significant increase in MDA concentration in the prefrontal cortex (nicotine + mephedrone at the dose of 0.05 mg/kg—*p* < 0.001; nicotine + mephedrone at the dose of 2.5 mg/kg—*p* < 0.01) in comparison with appropriate mephedrone-treated group.

**Fig. 5:**
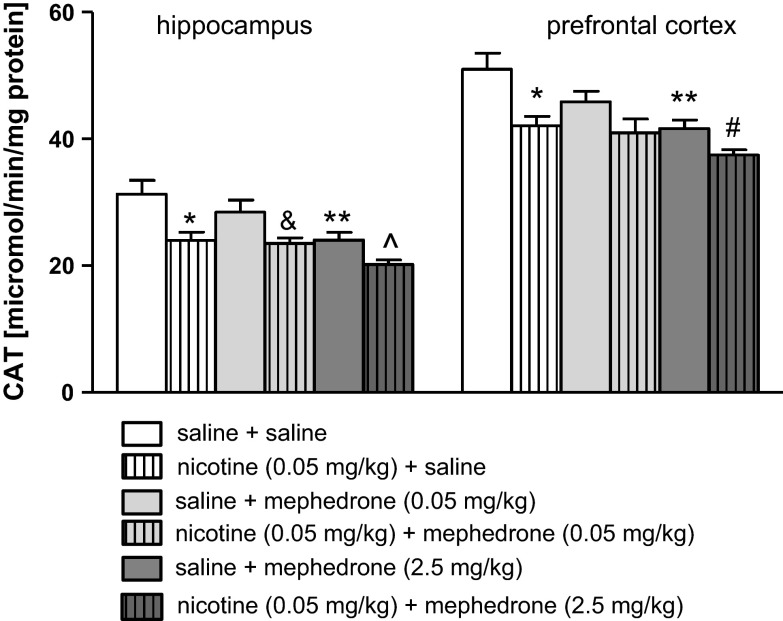
Effect of co-administration of mephedrone (0.05 or 2.5 mg/kg, i.p.) and nicotine (0.05 mg/kg, s.c.) on activity of CAT in the hippocampus and prefrontal cortex of mice. Data are presented as the mean ± SEM; n = 8–10; *p < 0.05; **p < 0.01 versus saline-treated control group; ^^^ *p* < 0.05 versus mephedrone 2.5 mg/kg-treated control group; ^&^ *p* < 0.05 versus mephedrone 0.05 mg/kg-treated control group; ^#^ *p* < 0.05 versus nicotine-treated control group; Tukey’s test

Figure 5 shows the activities of CAT after co-administration of mephedrone and nicotine in the hippocampus [two-way ANOVA: pre-treatment (F(1, 50) = 21.82, *p* < 0.0001), and treatment (F(2, 50) = 7.94, *p* = 0.0001), without interaction effect (F(2, 50) = 0.81, *p* = 0.4528)] and the prefrontal cortex [two-way ANOVA: pre-treatment (F(1, 50) = 16.14, *p* = 0.0002) and treatment (F(2, 50) = 7.36, *p* = 0.0017) without interaction effect (F(2, 50) = 0.97, *p* = 0.3851)]. The post hoc Tukey’s test revealed that an acute injection of nicotine at the dose of 0.05 mg/kg significantly decreased the activity of CAT in both examined structures (*p* < 0.05) versus saline-treated control group. Also, mephedrone (2.5 mg/kg) statistically decreased the activity of the enzyme in the hippocampus and prefrontal cortex (*p* < 0.01) (see also the same effects for the dose of 5 mg/kg, Supplementary Fig. 10). Moreover, co-administration of nicotine (0.05 mg/kg) and mephedrone (0.05 mg/kg) significantly deceased CAT activity in the hippocampus (*p* < 0.05) in comparison to mephedrone–treated group. Furthermore, significant decrease in CAT level was observed in the hippocampus when mephedrone (2.5 mg/kg) was co-administered with nicotine versus mephedrone-treated mice (*p* < 0.05) as well as in the prefrontal cortex (*p* < 0.05) versus nicotine-treated mice.

**Fig. 6:**
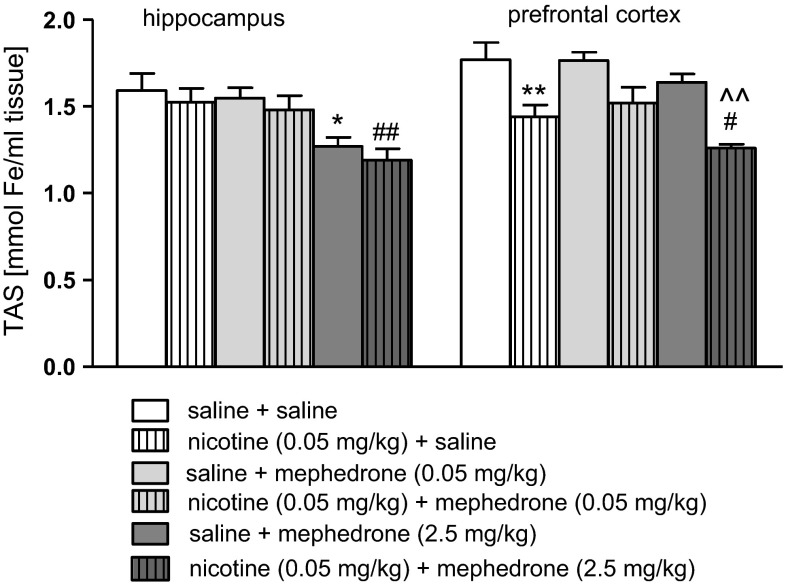
Effect of co-administration of mephedrone (0.05 or 2.5 mg/kg, i.p.) and nicotine (0.05 mg/kg, s.c.) on activity of TAS in the hippocampus and prefrontal cortex of mice. *p < 0.05; **p < 0.01 versus saline-treated control group; ^^^^ *p* < 0.01 versus mephedrone 2.5 mg/kg-treated control group; ^#^ *p* < 0.05; ^##^ *p* < 0.01 versus nicotine-treated control group; Tukey’s test

Figure 6 shows the values of TAS after co-administration of mephedrone and nicotine in the hippocampus [two-way ANOVA: treatment (F(2, 54) = 11.54, *p* < 0.0001) without pre-treatment (F(1, 54) = 1.46, *p* = 0,2300), and interaction effect (F(2, 54) = 0.01, *p* = 0.9960)] and the prefrontal cortex [two-way ANOVA: pre-treatment (F(1, 54) = 33.15, *p* < 0.0001) and treatment (F(2, 54) = 4.54, *p* = 0.0150) without interaction effect (F(2, 54) = 0.52, *p* = 0.5992)]. The post hoc Tukey’s test revealed that an acute injection of nicotine at the dose of 0.05 mg/kg significantly decreased TAS values in the prefrontal cortex (*p* < 0.01) versus saline-treated control group. Also, mephedrone (2.5 mg/kg) significantly decreased the concentration of antioxidants in the hippocampus (*p* < 0.05) (see also the same effects of the dose of 5 mg/kg in both structures, Supplementary Fig. 11). Moreover, co-administration of nicotine (0.05 mg/kg) and mephedrone (2.5 mg/kg) led to significant decrease in TAS value in the hippocampus (*p* < 0.01) and prefrontal cortex (*p* < 0.05) in comparison with nicotine-treated group. Furthermore, significant decrease in TAS level was also observed when mephedrone (2.5 mg/kg) was co-administered with nicotine in the prefrontal cortex (*p* < 0.01) versus mephedrone-treated mice.

## Discussion

This study attempts for the first time to show the acute, behavioral effects of mephedrone and nicotine co-administration using animal experimental models. In addition, we assessed the effect of the combined administration of mephedrone and nicotine on oxidative status in the brain. Our results showed that animals treated with mephedrone and nicotine at the subthreshold doses demonstrated elevated level of anxiety in the EPM test and displayed pro-cognitive behaviors in the PA paradigm. Also, the results confirmed that repeated daily injections of nicotine produced progressive increases in locomotor activity in mice, especially to a subsequent nicotine challenge. One of the main findings of the current study was that mephedrone, administered at the dose not influencing the locomotor activity of mice, enhanced the expression of nicotine-induced sensitization. Indeed, nicotine-experienced mice showed an increased response to the mephedrone injection compared with both the first pairing day and the response to acute mephedrone challenge in animals pre-exposed to saline. Additionally, our results indicated that mephedrone as well as nicotine exerted strong pro-oxidative effects. First, the research proved a decrease in total antioxidant status, i.e., general concentrations of all antioxidant molecules present in the samples of the hippocampus and the prefrontal cortex. Then, decrease in CAT activity confirmed that the oxidative action of the drugs was associated with overproduction of reactive oxygen species; here H_2_O_2_, as CAT is involved in hydrogen peroxide disintegration to water and oxygen. Finally, an increase in MDA concentration revealed induction of oxidative damages to brain lipids. Additionally, co-administration of both psychoactive substances intensified the oxidative changes in particular structures of the brain.

Many of the available studies indicate that amphetamines increase neuropsychiatric symptoms like anxiety in animal models as well as humans [34–37]. On the other hand, den Hollander et al. [38] observed that anxiety- and depression-related behaviors did not appear after mephedrone administration using binge like regimen. However, our studies revealed that this compound administered alone exerted a strong anxiogenic action in the dose range of 0.25–10 mg/kg, but the dose of 0.05 mg/kg did not influence the observed effect.

Our previous results have shown that a single injection of nicotine at the dose of 0.1 mg/kg had a significant anxiogenic effect in the EPM test [27], whereas the dose of 0.05 mg/kg of nicotine did not statistically change the values of time spent in open arms and number of entries into the enclosed arms of the maze [28]. In the present experiments, when mephedrone and nicotine were co-administered at the nonactive doses, a powerful anxiogenic effect was observed. Mice treated with both drugs spent less time in the open arms and presented a lower percentage of entries into enclosed arms of EPM test. Entries into enclosed arms increased only in groups treated with mephedrone alone at the doses of 2.5, 5 and 10 mg/kg, suggesting that motor effects are not the cause of the anxiogenic response.

The pathogenesis of anxiety is associated with deviation in release of different neurotransmitters, and engages a variety of brain structures. It is well established that neuronal system such as cholinergic, adrenergic, dopaminergic, GABA-ergic, serotoninergic and glutamatergic [12] play roles in anxiety processes. As stated previously, mephedrone increases extracellular DA, 5-HT and noradrenalin level in the CNS [4, 5]. Also, activation of the nAChRs by nicotine enhances the release of many neurotransmitters, including DA, ACh, noradrenalin, glutamate and 5-HT involved in the anxiety modulation of drugs [12]. We may suspect that enhancing of anxiety level observed in our experiments may be dependent on severity of monoamine transmission after co-administration of both drugs. However, on the basis of our biochemical studies we may also suggest that anxiety-like behaviors are not associated with oxidative damage in the CNS. Co-administration of low doses of both drugs increased only MDA level in the prefrontal cortex and decreased CAT activity in the hippocampus without influence on the other measured parameters.

Moreover, very strong anxiogenic activity of mephedrone was observed in the EMP test. 50-fold and tenfold lower doses (0.1 and 0.25 mg/kg) were active in this test than in the PA paradigm. We can not exclude that this difference in the effectiveness may be due to a local non-specific irritant effect of administered mephedrone by intraperitoneal injections. It is known that repeated intravenous injections of mephedrone in humans (even only 2–3 times) lead to local necrotic lesions. For this reason, human administration is normally oral, nasal or rectal [1–3].

Another aim of the present research was to record the interaction between mephedrone and nicotine at the level of cognitive processes at the consolidation trial, using the PA test in mice. Evidences show that memory deficits occurs after chronic use of psychostimulants, and the neurodegenerative effects of these compounds may lead to cognitive disabilities [39, 40]. Recent research also indicates that chronic or subchronic administration of mephedrone causes a reduction in memory function in rats [7, 38, 41]. However, other data indicate that mephedrone increases cognitive function after an acute administration similarly to other stimulants [42]. Also, series of study show that a post-training injection of d-amphetamine improves memory storage processes in the consolidation phase [43]. Our studies correspond with these results, as an acute administration of mephedrone, at the highest dose (5 mg/kg), improved memory processes in the consolidation trial. In animals, step-through latency to enter the dark compartment was significantly longer in the 24 h test, showing that they remembered the training session. Furthermore, in the current study we revealed that co-administration of subthreshold doses of mephedrone and nicotine produced an improvement in the memory processes in animals during the consolidation phase.

It is also well known that nicotine induces cognitive effects [44–46]. According to the previous study, we chose the subthreshold dose of nicotine (0.05 mg/kg) which has not affected memory and learning processes [28]. It should be noted that experiments with post-training drug administration have provided strong evidence that the memory enhancing effect of the drug is not a consequence of influences on acquisition processes or performance, because rodents are drug-free during the pre-test and test [47, 48]. Moreover, when the consolidation processes are measured, the stimulation of locomotor activity by mephedrone at the dose 5 mg/kg does not affect the obtained result.

Two anatomic structures have been recognized as important players in memory consolidation, e.g., the hippocampus and neocortex. These processes are mediated mainly by NMDA receptor activation and by dopaminergic systems [49, 50]. Additionally, GABA, noradrenalin, and 5-HT are also involved in the memory modulation [12, 51]. The neurobiological mechanisms of cognitive-enhancement by nicotine are well-characterized, and among all central nAChR subtypes, both the α4β2 combination and the α7 subunits appear to play important roles in memory-related responses [52]. The prefrontal cortex and the hippocampus seem to be important target sites for the nicotine effects on memory function [53, 54]. It is possible to suggest that the synergistic effect of an acute administration of nicotine and mephedrone on cognitive processes observed in our experiments may be explained by the influence of both drugs on dopaminergic and serotoninergic neurotransmission in the brain. Unfortunately, previously mentioned brain structures, e.g., hippocampus and prefrontal cortex are very susceptible to oxidative damage. Generally, a lot of evidence exists that intensification of oxidative processes is the primary cause of neurodegeneration. Indeed, mephedrone administered alone at the higher doses (2.5 and 5 mg/kg) as well as co-administration of mephedrone and nicotine induced oxidative stress observed as an increase in MDA levels and a decrease in TAS values and CAT activity in both structures. Therefore, we could expect impairment of memory processes. However, mephedrone-induced oxidative stress may not be long lasting, taking into consideration strong defense mechanisms of healthy organisms of experimental animals, as in the experiment only single doses of the drug were used. It shows proper functioning of an antioxidant system, which consists of low-molecular weight endogenous antioxidants, antioxidant proteins and enzymes as well as many regulator proteins that mediate adaptive responses to oxidative stress [55]. Therefore, such a one-time pro-oxidative intervention may stimulate antioxidant barrier to enhance release of intracellular antioxidants as well as up-regulation of ROS-metabolizing enzymes expression to counteract similar episodes in future. In our experiments, the memory processes were investigated 24 h after drug injection, when we did not observe changes in antioxidant status of brain tissue (data not shown). The attempts to understand the mechanisms underlying adaptive cell responses to mephedrone as well as nicotine-induced oxidative stress will provide new insight into development of neuroprotective treatment among psychoactive drug users and effective strategies for withdrawal therapy.

Furthermore, behavioral sensitization observed in our experiments, refers to a phenomenon by which the repeated use of a drug produces a progressive increase in the psychomotor response and has been implicated in the development of drug addiction [56–59] and drug-induced psychosis [60]. Behavioral sensitization can persist for several weeks and results from neuroplasticity in mesolimbic dopaminergic pathways [58, 61, 62]. Several drugs have been found to induce behavioral sensitization including cocaine, amphetamine, opiates [62] and nicotine [63]. It has been shown that repeated treatment with an addictive drug produces cross-sensitization defined as hyper-responsiveness to one psychostimulant after pre-exposure to a different drug [56, 64–66]. We used the nicotine-induced locomotor sensitization procedure to examine if nicotine-experienced mice show hyperactivity after an acute mephedrone administration. Our results confirmed that repeated daily injections of nicotine produced progressive increases in locomotor activity in mice, especially to a subsequent nicotine challenge [35, 67, 68]. Experimental data show that the expression of both acute and sensitizing locomotor effects of nicotine is coincident with functional changes in mesolimbic dopaminergic neurotransmission [69]. Concerning mephedrone, this drug induces short-term hyperlocomotion due to increased DA and endogenous 5-HT levels [4, 26, 70]. One of the main findings of the present study was the development of locomotor hyperactivity to mephedrone in nicotine-sensitized mice. We can’t exclude the existence of the full cross-sensitization phenomenon between nicotine and mephedrone, due to their similar neural pathways affected, but studies concerning the ability of mephedrone to elucidate behavioral sensitization are still unexplored and further analysis are needed.

In summary, abuse of psychoactive drugs constitutes a major problem worldwide, especially among young adults. The structural similarities of methcathinone and methylone with mephedrone suggest that we may still not appreciate the long-term risks of mephedrone use. There is also a great tendency for drug users to engage in simultaneous poly-substance use. Thus, polydrug use may be the rule rather than exception. Our study has shown the existence of strong interaction after an acute, concomitant administration of nicotine and mephedrone in anxiety, memory and locomotor sensitization experimental paradigms. Co-administration of subthreshold doses of both drugs exerts an anxiogenic effect and memory improvement. Both substances, mephedrone and nicotine, have been found to exert strong pro-oxidant effect on brain tissue. This influence was even stronger when both drugs were administered together. Finally, locomotor hyperactivity between nicotine and mephedrone when mice were pretreated with nicotine was obtained. Understanding the consequences of co-administration of psychoactive substances on the CNS and oxidative processes in the brain provide the toxicological significance, and may be useful in polydrug intoxication treatment. However, further research is required to determine long-term effects of mephedrone.

## Electronic supplementary material

Supplementary material 1 (DOC 371 kb)

Supplementary material 2 (DOC 30 kb)

Supplementary material 3 (DOC 29 kb)
