# Supplementary material for: Mephedrone and Nicotine: Oxidative Stress and Behavioral Interactions in Animal Models
Source: Neurochem Res. 2015 Apr 11;40(5):1083–93. doi: 10.1007/s11064-015-1566-5 (PMC4422847; doi:10.1007/s11064-015-1566-5)
Supplement: Supplementary file 1 — Supplementary material 1 (DOC 371 kb) [file 11064_2015_1566_MOESM1_ESM.doc]

Mephedrone and nicotine - oxidative stress and behavioral interactions in animal models

Barbara Budzynska, Anna Boguszewska-Czubara, Marta Kruk-Slomka, Jacek Kurzepa, Grazyna Biala

**Supplementary Figures 7-11**

**Figure 7**. Effects of an acute mephedrone or saline injection on the memory consolidation trial using the PA test in mice. Mephedrone (1, 2.5 and 5 mg/kg; i.p.) or saline were administered immediately after the pre-test; n=8-10; the means ± SEM; * p<0.05 *vs*. saline control group; Tukey’s test.

**Figure 8**. Effect of mephedrone (0.05-10 mg/kg) on spontaneous locomotor activity in mice. Locomotor activity (number of interruptions of light beams) was recorded immediately after injections of the drug for the 30 min. Data are presented as the means ± SEM; n=8-10; * p<0.05, ** p<0.01 *vs*. saline-treated control group, Tukey’s test.

**Figure 9.** Effect of administration of mephedrone (0.05-5 mg/kg, i.p.) on concentration of MDA in the hippocampus and prefrontal cortex of mice. Data are presented as the means ± SEM; n=8-10; * p<0.05, ** p<0.01 *vs*. saline-treated control group, Tukey’s test.

**Figure 10.** Effect of administration of mephedrone (0.05-5 mg/kg, i.p.) on activity of CAT in the hippocampus and prefrontal cortex of mice. Data are presented as the means ± SEM; n=8-10; * p<0.05, ** p<0.01 *vs*. saline-treated control group, Tukey’s test.

**Figure 11.** Effect of administration of mephedrone (0.05-5 mg/kg, i.p.) on activity of TAS in the hippocampus and prefrontal cortex of mice. Data are presented as the means ± SEM; n=8-10; * p<0.05, ** p<0.01 *vs*. saline-treated control group, Tukey’s test.
