# Supplementary material for: Mephedrone and Nicotine: Oxidative Stress and Behavioral Interactions in Animal Models
Source: Neurochem Res. 2015 Apr 11;40(5):1083–93. doi: 10.1007/s11064-015-1566-5 (PMC4422847; doi:10.1007/s11064-015-1566-5)
Supplement: Supplementary file 2 — Supplementary material 2 (DOC 30 kb) [file 11064_2015_1566_MOESM2_ESM.doc]

Mephedrone and nicotine - oxidative stress and behavioral interactions in animal models

Barbara Budzynska, Anna Boguszewska-Czubara, Marta Kruk-Slomka, Jacek Kurzepa, Grazyna Biala

**Supplementary Table 1**

| treatment | saline | MEPH 10 mg/kg | MEPH 5 mg/kg | MEPH 2.5 mg/kg | MEPH 1 mg/kg | MEPH 0.5 mg/kg | MEPH 0.25 mg/kg | MEPH 0.1 mg/kg | MEPH 0.05 mg/kg | F, p |
| --- | --- | --- | --- | --- | --- | --- | --- | --- | --- | --- |
| percentage time spent in open arms | 12.83  ±1.83 | 0.06  ±0.05 *** | 0.07  ±0.07 *** | 0.23  ±0.23 *** | 2.75  ±1.63 *** | 1.15  ±0.47 *** | 1.20  ±0.30 *** | 0.37  ±0.22 *** | 12.83  ±1.83 | F(8,78)=26.07  p=0.0001 |
| percentage open arms entries | 23.80  ±3.45 | 0.98  ±0.98 *** | 0.56  ±0.56 *** | 0.83  ±0.83 *** | 10.59  ±5.68 | 12.93  ±3.52 | 19.26  ±2.58 | 7.702  ±3.75 ** | 30.29  ±3.69 | F(8,78)=10.45  p=0.0001 |
| enclosed arm entries | 11.92  ±0.79 | 17.67  ±1.52 ** | 17.14  ±2.07 ** | 17.44  ±1.86 * | 12.71  ±0.86 | 10.75  ±0.8814 | 13.17  ±1.56 | 5.25  ±2.55 | 7.67  ±1.57 | F(8,78)=3.28  p=0.0026 |

Table 1. Mean (± SEM) percentage time spent in open arms, percentage open arms entries and enclosed arm entries in the EPM test in mice. Mephedrone (MEPH, 0.05, 0.1, 0.25, 0.5, 1, 2.5, 5 and 10 mg/kg) or saline were administered 15 min prior the EPM test; n=8-10; *** p<0.001, ** p<0.01, * p<0.05 *vs*. saline control group, Tukey’s test.
