# Supplementary material for: Mephedrone and Nicotine: Oxidative Stress and Behavioral Interactions in Animal Models
Source: Neurochem Res. 2015 Apr 11;40(5):1083–93. doi: 10.1007/s11064-015-1566-5 (PMC4422847; doi:10.1007/s11064-015-1566-5)
Supplement: Supplementary file 3 — Supplementary material 3 (DOC 29 kb) [file 11064_2015_1566_MOESM3_ESM.doc]

Mephedrone and nicotine - oxidative stress and behavioral interactions in animal models

Barbara Budzynska, Anna Boguszewska-Czubara, Marta Kruk-Slomka, Jacek Kurzepa, Grazyna Biala

**Supplementary Results**

**Effects of mephedrone on anxiety-related processes in the EPM test in mice.**

As shown in Table 1, mephedrone (when administered acutely 15 min prior to the EPM test) exerted a significant effect on the percentage of time spent on the open arms as well as the percentage of open arm entries. A post hoc Tukey’s analysis showed that mephedrone, at the doses of 0.1, 0.25, 0.5, 1, 2.5, 5 and 10 mg/kg, significantly decreased the percentage of time spent on the open arms (p<0.001). Moreover, mephedrone significantly decreased the percentage of open arm entries at the doses of 2.5, 5 and 10 mg/kg (p<0.001) and 0.1 mg/kg (p<0.01) thus indicating an anxiogenic effect vs. saline-treated mice. The doses of 0.05 mg/kg of mephedrone did not cause any effect in the EPM paradigm, thus it was chosen for the subsequent experiments.

The effect of mephedrone on enclosed arms entries is shown in Table 1 (one-way ANOVA (F(8,78)=3.28; p=0.0026). The post hoc Tukey’s test showed that mephedrone significantly increased locomotor activity when injected at the doses of 5 and 10 mg/kg (p<0.01) as well as 2.5 mg/kg (p<0.05) as compared with saline-treated mice. Moreover, mephedrone (0.05, 0.1, 0.25, 0.5 and 1 mg/kg) given acutely did not provoke any changes in the number of enclosed arm entries in the EPM test, thus causing no changes in the locomotor activity of animals. Also, co-administration of mephedrone and nicotine did not influenced locomotor activity of mice in this paradigm (data not shown).

**Effects of mephedrone on memory-related processes in the PA test in mice.**

One-way ANOVA revealed that, at the consolidation trial, an acute i.p. administration of mephedrone (1, 2.5 and 5 mg/kg) did not significantly change IL values (one-way ANOVA F(3,35)=2.539; p=0.0739). However, the post hoc Tukey’s test showed that mephedrone, at the dose of 5 mg/kg, significantly increased IL as compared with saline-treated mice, indicating that the drug, at the used dose, improved memory and learning processes (p<0.05) (Fig. 7). The non-active dose of mephedrone (2.5 mg/kg) was then chosen to study the interactions of nicotine and mephedrone in cognitive processes in the PA test.

**Effects of mephedrone on locomotor activity in mice.**

Locomotor activity of mice was measured with photoresistor actimeters (circular cages, diameter 25 cm, two light beams). The animals were placed individually in an actimeter for 60 min. The number of crossings the light beams by the mice was recorded as the locomotor activity after 30 and 60 min. In order to measure locomotor effects of mephedrone (0.05, 0.1, 0.25, 1, 2.5, 5 and 10 mg/kg, i.p.), animals, naive for any drug treatment, were injected with the drug and immediately placed in the activity chamber. Locomotor activity, i.e., the number of photocell beam breaks was automatically recorded.

The effect of mephedrone on locomotor activity in mice is shown in Figure 8 (one-way ANOVA (F(8,66)=4.5930, p=0.0002). The post hoc Tukey’s test showed that mephedrone significantly increased locomotor activity at the doses of 2.5 and 10 mg/kg (p<0.01) and 5 mg/kg (p<0.05) as compared with saline-treated mice. Moreover, mephedrone (0.05 – 1 mg/kg) given acutely did not caused changes in the locomotor activity of animals.

**The effect of mephedrone on oxidative stress biomarkers**

To determine whether mephedrone could induce the oxidative stress in vivo, we first examined the level of MDA, then activity of CAT and values of TAS in the tissues homogenates in the hippocampus and the prefrontal cortex.

Mephedrone increased the level of MDA [one way ANOVA: hippocampus (F(4,48)=5.875, p<0.0001); prefrontal cortex (F(4,48)=8.293, p<0.0001)]. The post hoc Tukey’s test showed that a significant increase in activity of MDA in the hippocampus and prefrontal cortex was observed in the group administered with mephedrone at the doses of 2.5 and 5 mg/kg (p<0.05, p<0.01, respectively) (Fig. 9).

A significant decrease in the activity of CAT [one way ANOVA: hippocampus (F(4,46)=4.241, p<0.0057); prefrontal cortex (F(4,46)=4.582, p<0.0038)] were found in the mephedrone-treated groups. Indeed, the post hoc Tukey’s test revealed that administration of mephedrone at the doses of 2.5 and 5 mg/kg statistically significant decreased CAT level in comparison to saline-treated group in the hippocampus (p<0.05 – both doses) as well as prefrontal cortex (2.5 mg/kg - p<0.05; 5 mg/kg - p<0.01) (Fig. 10).

A significant decrease in the TAS level [one way ANOVA: hippocampus (F(4,48)=4.930, p<0.0023); prefrontal cortex (F(4,48)=7.458, p<0.1136)] were found in the mephedrone-treated groups. Indeed, in the hippocampus isolated from the mephedrone-treated group, TAS level was significantly lower than that in the control group (2.5 mg/kg - p<0.05; 5 mg/kg - p<0.01; post hoc Tukey’s test). Furthermore, the injection of mephedrone at the dose of 5 mg/kg led to the decrease in TAS values (p<0.05) as compared with the control group in the prefrontal cortex (Fig. 11).
